# Characteristics of the spinopelvic parameters of patients with sacroiliac joint pain

**DOI:** 10.1038/s41598-021-84737-1

**Published:** 2021-03-04

**Authors:** Juichi Tonosu, Hiroyuki Oka, Kenichi Watanabe, Hiroaki Abe, Akiro Higashikawa, Takuya Kawai, Koji Yamada, Hiroyuki Nakarai, Sakae Tanaka, Ko Matsudaira

**Affiliations:** 1 Department of Orthopedic Surgery, Kanto Rosai Hospital, 1-1, Kidukisumiyoshicho, Nakahara-ku, Kawasaki, Kanagawa 211-8510 Japan; 2 Department of Medical Research and Management for Musculoskeletal Pain, 22nd Century Medical and Research Center, Faculty of Medicine, The University of Tokyo, Tokyo, Japan; 3 Department of Orthopedic Surgery, Faculty of Medicine, The University of Tokyo, Tokyo, Japan

**Keywords:** Pain, Radiography, Musculoskeletal system, Bone

## Abstract

To evaluate the characteristics of the spinopelvic parameters on radiography in patients with sacroiliac joint pain (SIJP). Two hundred fifty patients were included and divided into the SIJP group (those diagnosed with SIJP based on physical findings and response to analgesic periarticular injections; n = 53) and the non-SIJP group (those with low back pain [LBP] because of other reasons; n = 197). We compared their demographic characteristics and spinopelvic parameters using radiography. All differences found in the patients’ demographic characteristics and spinopelvic parameters were analyzed. More female participants experienced SIJP than male participants (P = 0.0179). Univariate analyses revealed significant differences in pelvic incidence (PI) (P = 0.0122), sacral slope (SS) (P = 0.0034), and lumbar lordosis (LL) (P = 0.0078) between the groups. The detection powers for PI, SS, and LL were 0.71, 0.84, and 0.66, respectively. Logistic regression analyses, after adjustment for age and sex, revealed significant differences in PI (P = 0.0308) and SS (P = 0.0153) between the groups, with odds ratios of 1.03 and 1.05, respectively. More female participants experienced SIJP than male participants. Higher PI and SS values were related to SIJP among LBP patients.

## Introduction

The sacroiliac joint (SIJ) supports the upper body when walking or standing and is not very mobile. Notably, the SIJ is one of the common origins of low back pain (LBP)^1–3^. Although LBP originating from SIJ is regarded as one of the non-specific causes of LBP^4^, some reports suggest that SIJ pain (SIJP) accounts for 10–30% of all cases of LBP^2,3^. There are no specific computed tomography (CT) findings for the diagnosis of SIJP. Degenerative findings around the SIJ were found to be highly prevalent in the asymptomatic population^5^. A study demonstrated that a substantial proportion of healthy active individuals without any symptoms also displayed bone marrow edema lesions around SIJ on magnetic resonance imaging (MRI)^6^. These studies indicate a lack of consensus regarding the effectiveness of diagnosing SIJP using both CT and MRI. Therefore, a diagnosis should be made by obtaining the results of pain provocation tests and by determining whether relief can be achieved by analgesic block injection to the SIJ^1–3^. Widely used provocation tests such as Gaenslen’s, Patrick’s, thigh thrust, compression, and distraction tests do not show high specificity^7,8^. The SIJ scoring system consists of physical findings and has been developed and validated as a diagnostic tool of SIJP^9,10^. The tool is useful for screening, but not for definite diagnosis. Therefore, the diagnosis of SIJP remains challenging.

Legaye et al.^11^ introduced the concept of pelvic incidence (PI) as a fundamental pelvic parameter of spinal sagittal curves in 1998. They reported that the PI value was equal to the sum of the sacral slope (SS) and pelvic tilt (PT). A previous study that investigated characteristics of spinopelvic parameters for patients with SIJP following lumbar surgery demonstrated that higher PI values were significantly associated with SIJP^12^. However, patients with SIJP following lumbar surgery might have had the potential to develop SIJP even if they had not undergone surgery because PI was a specified value for each person in any situation^11^.

Reports that have examined the relationship between cases of SIJP and spinopelvic parameters are lacking. This led us to hypothesize that higher PI values were related to SIJP. The purpose of the current study was to evaluate the characteristics of the spinopelvic parameters on radiography in SIJP patients.

## Methods

### Subjects

The study was performed at one of the core hospitals in our city. The hospital has surgical and out-patient departments. The out-patient department adopts a complete referral system; therefore, only patients who have a referral letter go to the hospital for consultation. A total of 281 consecutive LBP patients were registered consecutive LBP patients from June 2015 to December 2019 during their first visit. LBP was defined as a pain localized between the costal margin and the inferior gluteal folds. Radiography of the lumbar spine and pelvis at a standing position were evaluated for all patients. Lumbar MRI examinations were added and evaluated for LBP cases with buttock and/or lower limb pain as well as severe LBP cases without buttock or lower limb pain. Following these radiological evaluations, patients who had an infection or tumor in the lumbar spine or pelvis were excluded. We excluded 24 cases of metastatic lumbar spine tumor and 7 cases of lumbar infectious spondylitis/discitis. Finally, a total of 250 patients were included in the study. Subsequently, an analgesic periarticular SIJ injection was administered in order to diagnose and evaluate patients who indicated buttock pain in the region from the posterior iliac crest to the inferior gluteal fold and with positive result on either the Patrick’s test or the SIJ shear test. We defined the SIJP group (n = 53) as those who experienced unilateral buttock pain and fulfilled the criteria described here, as defined in our previous report^10^. The non-SIJP group (n = 197) was defined as those who had experienced LBP due to reasons other than SIJP. We used a self-written questionnaire to collect patient background information that included age, sex, height, weight, and smoking habits. The body mass index (BMI) was calculated using height and weight data. The first author measured spinopelvic parameters such as PI, PT, SS, and lumbar lordosis (LL) using the radiographs. We compared and analyzed the demographic characteristics and spinopelvic parameters of both groups. The medical/ethics review board of Kanto Rosai Hospital approved the study, including any relevant details, and confirmed that all experiments were performed in accordance with relevant guidelines and regulations and that informed consent was obtained from all participants.

### Definition of SIJP

We used our previously published definition for SIJP^10^. We precisely defined and evaluated the region where buttock pain occurred as pain at the area from the posterior iliac crest to the inferior gluteal fold^10^. We defined SIJP as unilateral buttock pain that met the following criteria: positive results on either the Patrick’s test or the SIJ shear test, which are direct stress tests of the SIJ region^1^; positive response to the analgesic periarticular SIJ injection; no findings of nerve root or cauda equina compression on lumbar MRI; no hip osteoarthritis on radiography of the hip. We evaluated physical findings, including Patrick’s and SIJ shear test results, and radiographs of the lumbar spine and pelvis. When we suspected SIJP, we evaluated lumbar MRI findings in order to rule out the possibility of lumbar diseases and performed an additional examination using an analgesic SIJ injection, which was a 3-mL injection of 1% lidocaine, into the periarticular space of the SIJ with fluoroscopic control in reference to previous studies^3,8^. We evaluated the degree of buttock pain both before and after the injection. We considered a reaction to the analgesic injection as positive when the pain had improved more than 70% 15 min after the injection.

### Statistical methods

Descriptive statistics were determined and presented. Using the Anderson–Darling test, we evaluated whether each item was normally distributed or not. Between-group differences in demographic characteristics were evaluated using the Fisher’s exact test for categorical variables. Continuous variables were shown as means ± standard deviation for parametric items and medians as and first/third quantiles for non-parametric items. The P values were calculated by Student's t-test and the Wilcoxon rank sum test for parametric and non-parametric items, respectively. The post hoc detection power was calculated as 1 − beta and represents the probability that we reject the null hypothesis when it was false^13^. In addition, we performed logistic regression analyses adjusted by age and sex for the spinopelvic parameters. One guideline suggested that accurate estimation of the discriminant function parameters required a sample size of at least 20 cases for each independent variable^14^. The number of the case group was 53; thus, three variables, including age and sex, were adopted in the logistic regression analyses for each parameter. Correlation coefficient analysis was performed for each of the spinopelvic parameter items. We performed receiver operating characteristic (ROC) curve analysis and calculated the area under the ROC curve (AUC), sensitivity, and specificity for each item. Statistical analysis was performed using JMP version 14.0 (SAS Institute, Cary, NC, USA). A P value < 0.05 was considered significant.

## Results

There were no significant differences in the demographic characteristics (age, BMI, and smoking habits) between the groups except for sex. The ratio of female participants was significantly higher in the SIJP group than that in the non-SIJP group (P = 0.0179). Among the continuous variables, PI and SS were normally distributed, whereas age, BMI, PT, and LL were not normally distributed. There were significant differences in PI (P = 0.0122), SS (P = 0.0034), and LL (P = 0.0078) between the groups (Table 1). The detection powers for PI, SS, and LL were 0.71, 0.84, and 0.66, respectively. The mean PI values were significantly higher for female than for male participants (51.6° ± 12.0° vs. 47.9° ± 9.3°; P = 0.0089). The mean SS values for female and male participants were not significantly different (34.0° ± 9.5° vs. 33.0° ± 8.1°; P = 0.3789). Logistic regression analyses of 250 radiographs, after an adjustment for age and sex, revealed significant differences in PI (P = 0.0308) and SS (P = 0.0153) between the groups, with odds ratios of 1.03 and 1.05, respectively (Table 2). Among each of the spinopelvic parameters, SS and LL showed a strong correlation coefficient of 0.764, followed by a correlation coefficient of 0.676 for PI and SS (Table 3). The ROC analyses demonstrated that the AUC, cutoff, sensitivity, and specificity values were 0.60971, 50, 0.660, and 0.538, respectively, for PI, and 0.63006, 38, 0.509, and 0.736, respectively, for SS.

**Table 1:** Demographic characteristics and spinopelvic parameters by univariate analyses.

	SIJP (+) (n = 53)	SIJP (−) (n = 197)	P value
Age (years)	50 [41.5, 62.5]	56 [45, 69.5]	0.0860
Sex (male/female)	14/39	89/108	0.0179*
Body mass index (kg/m^2^)	22.9 [20.2, 25.1]	21.8 [20.1, 24.3]	0.2360
Smoking (+ /−)	9/44	41/155	0.6992
Pelvic incidence (°)	53.5 ± 12.6	49.2 ± 10.6	0.0122*
Pelvic tilt (°)	17 [10, 21.5]	15 [10, 21.5]	0.7110
Sacral slope (°)	36.7 ± 9.1	32.7 ± 8.7	0.0034*
Lumbar lordosis (°)	50 [42, 55.5]	44 [36, 53]	0.0078*

Data are presented as means ± standard deviation for parametric items and medians and first/third quantiles for non-parametric items, or as numbers of participants (%).

*SIJP* sacroiliac joint-related pain.

*P < 0.05. The P values were calculated by Student's t-test and the Wilcoxon rank sum test for parametric and non-parametric items, respectively.

**Table 2:** Logistic regression analyses for odds ratios and 95% confidence intervals of the spinopelvic parameters for sacroiliac joint-related pain.

Item	Odds ratio	95% Confidence interval	P value
Pelvic incidence	1.03	1.003–1.061	0.0308*
Pelvic tilt	1.01	0.969–1.048	0.6928
Sacral slope	1.05	1.009–1.087	0.0153*
Lumbar lordosis	1.02	0.997–1.046	0.0827

Data are calculated by logistic regression analysis after adjustment for age and sex on 250 radiographs.

The number of the case group was 53; thus, three variables, including age and sex, were adopted in the logistic regression analyses for each parameter.

*P < 0.05.

**Table 3:** Correlation coefficient of each item derived from the univariate analyses.

	Pelvic incidence	Pelvic tilt	Sacral slope	Lumbar lordosis
Pelvic incidence	–			
Pelvic tilt	0.611	–		
Sacral slope	0.676	− 0.171	–	
Lumbar lordosis	0.402	− 0.284	0.764	–

## Discussion

Higher PI and SS values were significantly related to SIJP. We defined SIJP in the study as unilateral buttock pain that met the following criteria: positive results on direct stress tests of the SIJ region^1^; positive response to the analgesic periarticular SIJ injection; no other lumbar spine or hip diseases.

The diagnosis of SIJP has not been clearly confirmed and remains challenging^1–3^. Laslett^15^ demonstrated that three or more positive pain provocation SIJ tests have sensitivity and specificity of 91% and 78%, respectively. However, other studies showed that widely used provocation tests do not show high specificity^7,8^. Dreyfuss et al.^16^ concluded that SIJP is difficult to identify using historical and physical examination data. Although these studies were published more than 10 years ago, the diagnosis of SIJP using only physical examination has remained challenging. Therefore, we did not consider several physical examinations as gold standard for diagnosing SIJP in our study; instead; therefore, we defined SIJP in detail as aforementioned. A literature review recommended using the term “pelvic girdle pain,” which included pain originating from the intraarticular region and the posterior ligamentous region of the SIJ^3^. Other studies have demonstrated that the posterior ligament region is a significant source of SIJP^17,18^. We had also adopted the same criteria using periarticular block for SIJP following lumbar spine surgery in our previous study^12^. The incidence was also as appropriate and acceptable as those reported in previous works regarding SIJP following lumbar spine surgery. Therefore, we adopted periarticular SIJ injection to evaluate the response to analgesic injections. We met the criteria using lumbar MRI and hip radiography for all patients in the SIJ group for exclusion of lumbar and hip causes, respectively. Although the lumbar MRI findings always reveal the causes of LBP, we performed lumbar MRI in our work to exclude specific findings, which could be associated with buttock pain development, such as lumbar disc herniation or lumbar spinal stenosis. Therefore, our definition of SIJP is appropriate.

The SIJP group included approximately 20% of all patients in the current study, which were consistent with those of previous studies that reported 10–30% in their SIJP groups^2,3^. No significant differences were observed regarding the demographic characteristics of the two groups except for sex. An epidemiological report showed that more female participants presented with SIJP than male participants^19^; however, reports about sex differences are scarce. Our result was consistent with this report and provides findings about sex differences related to SIJP. A study reported that the ligaments around the SIJ in female participants were not as strong as the ligaments in male participants, which could cause instability of the SIJ^20^. A finite element study showed that female participants diagnosed with SIJP had higher mobility, stresses, loads, and pelvis ligament strains than male participants, which could account for the higher incidence of SIJP in female participants^21^.

In the logistic regression analyses after adjustment for age and sex, we found that PI and SS were the significant items for the diagnosis of SIJP. A study showed that high PI and SS were related to LBP, especially in female participants^22^, whereas another report showed that low PI, SS, and LL were related to LBP in general^23^. Although both studies did not define the area of the LBP in detail and mentioned only SIJP, our result corresponded with the findings of the former study. PI is a sum of the PT and SS. In the current study, the PI and SS values were moderately correlated, and the difference in the PI values between the two groups could be attributed to that of the SS values, whereas there was not much difference in the PT values between the two groups.

A study showed that a posterior shift in the shearing force line that resulted from a high PI might place a load on the posterior components of the low back, which was followed by an increase in LBP^24^. That mechanism may account for our results; however, the PI value of the SIJP group in our study was similar to the Japanese normative value of 52.3°^25^. Therefore, our results could be interpreted as a low PI value of the non-SIJP group, rather than the value for the SIJP group being high. A small LL is often seen in patients with lumbar diseases such as spinopelvic imbalance^26^. Although the LL value was excluded after the logistic regression analyses after adjustment for age and sex, the LL value was highly correlated with the SS value and moderately correlated with the PI value in the current study. Therefore, the small SS and PI values could correlate with the small LL value in the non-SIJP group, in which the patients did not experience SIJP but rather LBP caused by spinopelvic imbalance. As the result, the SIJP group demonstrated significantly higher PI values than the non-SIJP group, which indicated that buttock pain without spinopelvic imbalance or findings of nerve root or cauda equina compression on lumbar MRI could indicate SIJP.

There were some limitations to this study. First, there could be false-positive responses to the analgesic injection. A false-positive response rate to the analgesic SIJ injection was found to be as high as 10–20%^8^. This limitation could result in an overestimation in the SIJP group. Second, the SIJP group may be underestimated depending on interpretation of lumbar MRI examinations. Asymptomatic lumbar disc herniation and lumbar spinal stenosis findings sometimes occur^27^. Moreover, a report demonstrated that SIJP often co-existed with other lumbar diseases^28^. Therefore, we could have excluded a possible cause of SIJP. Third, there was selection bias among our patients. Patients from one hospital cannot represent all patients with SIJP. However, an advantage of our study is that all consecutive patients seeking care for low back or buttock pain who met study eligibility were completely evaluated, including all the required examinations, tests, and imaging, which could decrease selection bias.

In conclusion, our study found that more female participants presented with SIJP than male participants. This study is the first to identify that higher PI and SS values were significantly related to SIJP among LBP patients. These results could be of value in diagnosing SIJP.

## Data Availability

The datasets generated during and/or analyzed during the current study are available from the corresponding author on reasonable request.
